# Enhanced Brain Connectivity Following Six Weeks of Upper Extremity Offset Loading in Neurotypical Adults—A Preliminary Study

**DOI:** 10.3390/s26123805

**Published:** 2026-06-15

**Authors:** Kayode Ahmed, Jessica M. Kirschmann, Erin S. Herder, Reedah F. Memon, Snehi B. Shah, Komal K. Kukkar, David Walsh, Craig A. Johnston, Pranav J. Parikh

**Affiliations:** 1 Department of Clinical Research, University of Jamestown, Jamestown, ND 58405, USA; 2 Department of Health and Human Performance, University of Houston, Houston, TX 77204, USA; jmkirsch@cougarnet.uh.edu (J.M.K.); emshier@central.uh.edu (E.S.H.); rfmemon@cougarnet.uh.edu (R.F.M.); sbshah5@cougarnet.uh.edu (S.B.S.); kkkukkar@cougarnet.uh.edu (K.K.K.); dwwalsh@central.uh.edu (D.W.); cajohn25@central.uh.edu (C.A.J.); pjparikh2@uh.edu (P.J.P.); 3 Department of CNS Radiation Oncology, The University of Texas MD Anderson Cancer Center, Houston, TX 77030, USA

**Keywords:** offset loading, resistance training, functional connectivity, cortical plasticity, sensorimotor integration, electroencephalography (EEG)

## Abstract

Symmetrical resistance training induces corticospinal and cortical network plasticity; however, the neural consequences of asymmetrical resistance training (offset loading or OL) remain unclear. In this preliminary study, fourteen healthy adults completed eighteen supervised upper-extremity training sessions over a 6-week intervention period. Participants were allocated to either an offset loading (OL) intervention group (*n* = 8) or a conventional symmetrical resistance training active control group (AC; *n* = 6). Electroencephalography (EEG) recordings were acquired during motor task performance at baseline, 3 weeks, and 6 weeks. Directed functional connectivity among predefined cortical regions was quantified, and longitudinal changes were assessed using Friedman tests with false-discovery-rate correction for multiple comparisons. Significant changes in the OL group were observed involving two cortical pathways after correction for multiple testing. Connectivity from the right parietal cortex to the right sensorimotor cortex increased over time (Friedman χ^2^ = 8.00, *q* = 0.037), with post hoc analyses showing a significant increase between the midpoint and post-training assessments (*q* = 0.047; effect size r = 0.894). No significant longitudinal changes in cortical connectivity were identified in the AC group. Six weeks of OL training was associated with selective strengthening of directed connectivity within prefrontal-to-sensorimotor and parietal-to-sensorimotor cortical pathways. In contrast, conventional symmetrical resistance training was not associated with detectable changes in connectivity. These findings suggest that asymmetrical loading may induce task-specific reorganization of cortical networks involved in sensorimotor processing and motor control.

## 1. Introduction

Resistance training (RT) is utilized to improve strength, enhance neuromuscular coordination, and promote functional autonomy [1,2,3]. Growing evidence in the literature suggests that RT promotes central adaptations in the brain and spinal cord that support motor learning, coordination, and executive function [4,5,6]. These adaptations are typified by reduced intracortical inhibition, increased cortical excitability, and efficient motor unit deployment, reflecting augmented neural drive and sensorimotor integration [4,7,8]. Direct electroencephalographic (EEG) evidence of supraspinal plasticity following resistance training has been demonstrated: three weeks of explosive unilateral leg extensor training attenuated movement-related cortical potential amplitude at motor-related scalp sites and shifted cortical potential onset earlier, reflecting enhanced neural economy at the level of the cortex [9]. Neuroimaging and electrophysiological studies reveal that these adaptive changes follow white-matter microstructure and cerebral perfusion alterations, including the coordinated activity of large-scale neural networks that modulate motor control and higher cognitive functions [10,11,12,13,14]. Evidence suggests that resistance exercise alters functional connectivity in premotor and prefrontal cortices, which are important for attention, inhibitory control, and motor planning [15]. These higher-order adaptations may contribute to improved cognitive regulation, including the capacity to select goal-directed actions [16]. The aforementioned neural effects are based on a resistance training paradigm where the same load is used to train both sides.

A novel asymmetrical resistance training regimen has been introduced, known as offset loading (OL), where a planned portion of a given training load is offset to one side, creating a loaded (heavier) side and a de-loaded (lighter) side. OL introduces contorted biomechanical demands that challenge proprioception and coordination, concomitantly involving additional neural circuits involved in sensorimotor integration [4,5,6]. The instability because of asymmetrical load requires higher-order attention monitoring to maintain postural equilibrium.

The offset loading technique leads to increased activation of muscles on the heavier side, and this may be due to increased descending neural drive to the muscles to manage load asymmetry. This may necessitate greater and widespread communication among sensorimotor brain regions and possibly the involvement of cognitive brain areas than traditional symmetrical loading for continued recalibration of muscle activation and posture [17]. EEG-based directed connectivity analysis during single-leg balancing on unstable surfaces has revealed that postural challenge establishes two topographically distinct cortical networks: a theta-band network spanning the frontal, central, and parietal cortices and an alpha-band network propagating from occipital to parietal and centro-parietal areas, demonstrating that complex balance demands recruit and functionally connect widespread cortical regions [18]. While symmetrical RT protocols cause task-specific reorganization and changes in functional connectivity in the brain, the neural effects of asymmetrical (offset) resistance training remain unexplored [19].

In this first mechanistic study, we investigated and compared the effects of six weeks of upper extremity training in the form of offset (asymmetrical) loading and symmetrical loading on functional connectivity between sensory, motor, and cognitive brain areas using electroencephalography in neurotypical adults. Functional connectivity between cortical regions is sensitive to training interventions, illustrating the ability of the brain to reconfigure functional networks in response to behavioral demands [2]. By integrating motor, sensory, and cognitive challenges, offset loading may promote cortical network reorganization. We hypothesize that offset loading will enhance the functional connectivity among cortical regions known to play a role in sensorimotor and cognitive behaviors. Furthermore, we hypothesize that these adaptations will reflect a task-specific neural strategy that differs from the patterns observed in traditional symmetrical loading.

## 2. Materials and Methods

### 2.1. Study Participants

Twenty-two healthy adults, aged 18 years or older, were recruited from the University of Houston community. Participants had a mean age of 21.5 ± 1.9 years (13 females). Most participants were right-handed, with one participant being left-handed. Mean height was 65.5 ± 3.5 inches, and mean body weight was 154.8 ± 30.0 lb. Participants were randomly assigned (1:1) to the offset loading (OL) and the symmetrical loading (active control; AC) groups. Of the twenty-two enrolled participants, fourteen completed the study. Participants who completed the study had a mean age of 21.5 ± 2.2 years, a mean height of 65.6 ± 3.7 inches, a mean body weight of 160.6 ± 33.4 lb, and a mean BMI of 26.0 ± 3.9 kg/m^2^. Demographic characteristics were generally similar between groups. The AC group had a slightly greater mean body weight (165.8 ± 40.5 lb) and BMI (26.2 ± 4.6 kg/m^2^) compared with the OL group (156.7 ± 29.3 lb and 25.9 ± 3.6 kg/m^2^, respectively). All participants were required to be right-handed, physically capable of performing the study procedures, willing to complete all components of the protocol, and free from any medical condition that might interfere with resistance exercise, particularly the chest press. Exclusion criteria included age under 18 years, inability to commit to the six-week training period, pregnancy, uncontrolled hypertension, a body-mass index (BMI) below 20 kg·m^−2^, or a musculoskeletal injury or disability affecting the upper limbs. Individuals who were unable to comprehend study procedures or English-language instructions were also excluded. Vulnerable populations, including minors, pregnant individuals, individuals unable to provide consent, and incarcerated persons, were excluded. Prospective participants completed a prescreening form before their initial laboratory visit. At the first visit, study staff reviewed eligibility criteria, obtained written informed consent, and administered physical activity questionnaires. Participants in the OL group reported high (*n* = 6), moderate (*n* = 1), and low (*n* = 1) levels of physical activity. Similarly, in the AC group, participants reported high (*n* = 3), moderate (*n* = 3), and low (*n* = 0) levels. Participation was voluntary, and a modest gift card was provided as compensation upon completion of the study. Eight participants in the OL group and six participants in the AC group completed all study procedures that included three assessment sessions and eighteen resistance training sessions. Data from these fourteen individuals were included for further analysis.

### 2.2. Instrumentation

**Figure 1:**
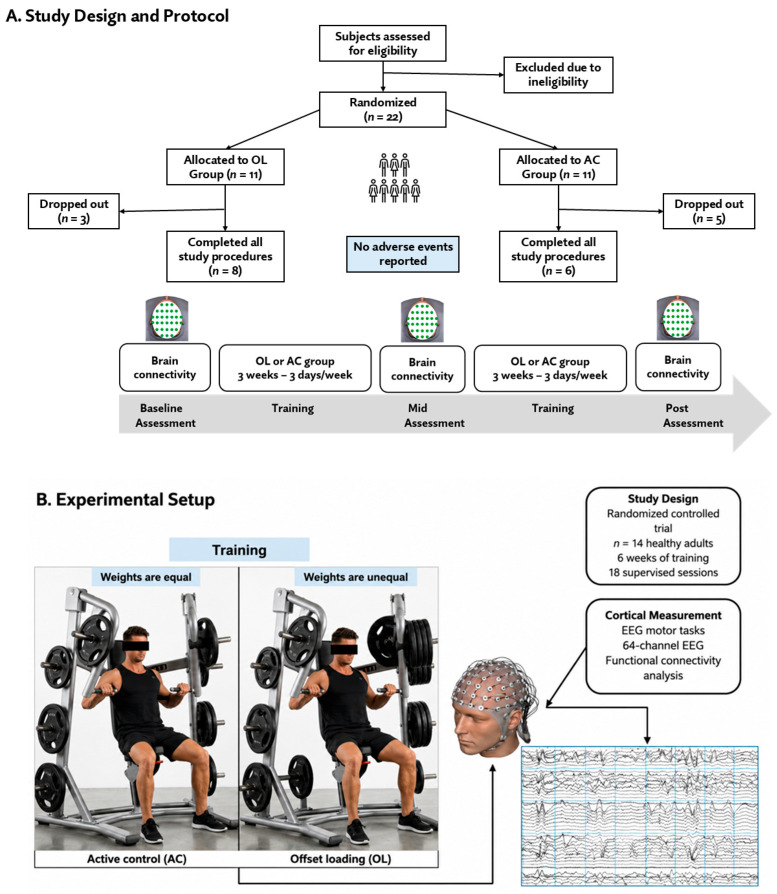
Study design, protocol and experimental setup. (**A**) shows the study design, participant recruitment and retention, group assignment, and timing of outcome assessments. (**B**) illustrates the experimental setup and intervention procedures for the offset-loading (OL) and active control (AC) groups, including training and testing configurations.

Electroencephalography (EEG): A whole-head EEG was acquired via 64 active channels (1000 Hz; Brain Products GmbH, Gilching, Germany) following the internationally recognized 10–20 system for EEG electrode positioning during chest press (Figure 1). Four electrodes were dedicated to the acquisition of electrooculographic (EOG) signals. To minimize movement-induced artifacts, particularly during the task, the EEG cable movement was restricted using an elastic mesh overlaid on the EEG cap [20,21]. Electrode impedances were maintained below 10 kΩ. A deviation from the standard international 10–20 EEG system was implemented by relocating the ground (GND) and reference [21] electrodes from their typical positions at AFz and FCz to the left and right earlobes, respectively. This adjustment was made due to the proximity of the conventional GND and REF locations to the cortical regions of interest. The T7 and T8 electrodes were repositioned to the AFz and FCz sites to compensate for the displaced positions, respectively.

Electromyography (EMG): Surface EMG was recorded from the biceps brachii, triceps brachii, and anterior deltoid muscles using bipolar Ag/AgCl electrodes (1111.11 Hz, gain 1000; 20–450 Hz Delsys Trigno EMG System, Boston, MA, USA). These muscles were selected because of their involvement in the control of the chest press. The EMG data are beyond the scope of this study and thus not reported in this manuscript, as the corticomuscular coupling analysis derived from these concurrent recordings addresses a distinct research question with separate methodological and interpretive considerations. Corticomuscular coupling analysis is being addressed in a forthcoming companion paper.

### 2.3. Resistance Training

Asymmetrical Loading (Offset Loading; OL): Participants in this group performed weightlifting with a plate-loaded iso-lateral chest press weightlifting machine for a total of six (6) weeks. They completed four sets, three days per week, with at least one day of rest between each training day. For each lifting set, individuals completed ten repetitions, followed by a brief rest period of ~1 min. The first two sets were paired to have the heavier load on one side, and the last two sets were paired to have the heavier load on the opposite side of the first set pair. The difference in load on the heavier and lighter side was 50%. The load on the heavier side will be set at 60% of 1RM [1]. The order (dominant arm or non-dominant arm) was randomized each day.

Symmetrical Loading (Active Control; AC): Participants performed weightlifting with a plate-loaded iso-lateral chest press for the same duration as the OL group (i.e., 6 weeks, 3 times per week, 4 sets, 10 repetitions per set). Different from the OL group, the AC group had equal amounts of weight distributed to both arms in all repetitions and sets. All other factors were held constant, outside of differing amounts of weight to the dominant or non-dominant arm.

### 2.4. Assessment

For this study, testing procedures were carried out at the University of Houston. Enrolled subjects completed standardized assessments across baseline (pre) assessment, mid-assessment, and post-assessment timepoints, to capture baseline and training adaptations. Baseline interventions captured at baseline (Week 0) or pre-intervention included electroencephalography (EEG) and electromyography (EMG). Repeated EEG and EMG assessments were performed at week 3 (mid-intervention assessment) to capture adaptations observed during training. The post-intervention assessment took place at Week 6 and consisted of a full repetition of all baseline measurements to quantify training-related changes over time. EEG and EMG were measured while participants performed the plate-loaded iso-lateral chest press with equal amounts of weight distributed to both arms in all repetitions and sets (4 sets, 10 repetitions per set, Figure 1).

### 2.5. Experimental Protocol

Participants in the training groups attended three supervised sessions per week for six weeks (18 sessions in total). Each session began with a standardized dynamic warm-up on a stationary bicycle. The load on the resistance chest press machine was adjusted to 60% of 1RM. To estimate 1RM, participants were instructed to perform the chest press through the full range of motion while gradually increasing the load. The 1RM was the maximum load a participant could lift for one single repetition through the full range of motion. The 1RM assessment was repeated every 2 weeks, and the loads in both training groups were adjusted accordingly. In the OL group, total workload matched that of the AC group but was distributed unequally between arms. EEG recordings were obtained during chest press during the Pre-, Mid-, and Post-intervention visits to assess brain connectivity. All exercise sessions were directly supervised by trained exercise specialists to ensure compliance and safety.

### 2.6. EEG Data Analyses

#### 2.6.1. Preprocessing

EEG pre-processing followed a standardized pipeline [22,23,24] consistent with the following steps: First, the continuous EEG data were downsampled to a sampling rate of 250 Hz. Ocular artifacts and slow signal drifts were removed using a robust H-infinity filter with parameters set to *γ* = 1.1 and *q* = 1 × 10^−11^. Eye-blink and eye-movement artifacts were further attenuated using electrooculogram (EOG) channels positioned around the eyes as reference signals. Line noise was eliminated using the Zapline function implemented in EEGLAB. Next, a standardized early-stage EEG processing pipeline (PREP) with default parameters was applied to identify and remove artifactual EEG channels using a common mean reference to enhance the signal-to-noise ratio. Signals from faulty measurement channels were replaced by interpolated data from functional neighboring censors to preclude skewing the re-referencing process. The data were band-pass filtered between 0.1 and 100 Hz using a fourth-order Butterworth filter to remove low-frequency drifts and high-frequency noise. Artifact Subspace Reconstruction (ASR) was subsequently used to detect and suppress high-amplitude artifact segments. A standard deviation threshold of 15 within a 500-ms moving window was applied using principal component analysis (PCA) to identify noisy data segments. Before performing Independent Component Analysis (ICA), surrogate channels generated by the PREP pipeline were removed to address the rank deficiency. The Adaptive Mixture Independent Component Analysis (AMICA) algorithm was used to conduct ICA in extracting maximally independent components. Data from all four sets (10 repetitions each) were included for each subject. The final EEG segments used for EEG analysis were constructed by concatenating the recording from each set, along with a 30-s window captured before and after the active contraction. The topological positions of the electrodes were aligned with the constructed head model. Each remaining component was visually inspected based on scalp topography, dipole location, and spectral power distribution to ensure the removal of residual non-brain activity. Clean EEG signals were reconstructed after independent components with dipoles located outside the BEM volume or associated with non-neural artifacts (e.g., muscle activity or motion-related noise) were excluded. After artifact removal, we had 134 ICs remaining for the OL group and 115 ICs remaining for the AC group.

#### 2.6.2. Granger Causality Connectivity

Before causality modeling, stationarity of each dipole time series was evaluated with the Augmented Dickey–Fuller test, a prerequisite for Granger causality analysis. The null hypothesis of a unit root was rejected for all time series (all test statistics < −79.4 and all *p* < 0.01), indicating that the time series were stationary. Granger causality was then calculated using a multivariate autoregressive (MVAR) modeling approach implemented with the MVGC Toolbox [25,26]. The MVGC Toolbox operationalizes G-causality through vector autoregressive (VAR) modeling and captures dependencies among multivariate time series. This modeling is well-suited for analyzing the dynamic interactions of neural signals, such as EEG data with high temporal resolution and stochastic nature [23]. The causality analysis in time series data estimates directed interactions. Consider two jointly distributed vector-valued stochastic processes X and Y. If the past of Y conveys information about the future of X above and beyond all information contained in the past of X, then we say that Y G-causes X. However, if there is no direct causal influence of Y on X, but there are dependencies of X and Y on another variable, say Z, then it can result in spurious Y on X causality. Pairwise-conditional G-causality was used to estimate the connectivity between two clusters while controlling for other identified clusters. This approach eliminates spurious causalities by ‘conditioning out’ the common dependencies if they are available in the data. The optimal model order, which determines the number of past time points used to predict future activity, was selected using the Akaike Information Criterion (AIC), resulting in a model order of 20. GC was computed bidirectionally between each two electrode pairs, allowing examination of both the influence of electrode X on electrode Y and vice versa. The GC analysis targeted four cortical regions, each represented by specific electrodes from the 10–20 system. The prefrontal region was defined by electrodes AF3 (left prefrontal) and AF4 (right prefrontal) and is associated with planning, executive control, and working memory. The parietal region, represented by P3 (left parietal) and P4 (right parietal), is involved in motor coordination and sensory integration. The central region, defined by C3 (left sensorimotor) and C4 (right sensorimotor), is important for motor execution and somatosensory processing. We considered bidirectional connections between AF3, C3, and P3 electrodes to assess the left hemisphere functional connectivity and between AF4, C4, and P4 to assess the right hemisphere functional connectivity. Bidirectional connectivity analysis between C3 and C4 allowed us to capture the interhemispheric connectivity. This ROI-based approach allowed physiologically interpretable measures of directional information flow between cortical areas. The GC analysis was run on the data from each assessment set and then averaged across four sets. Statistical significance was determined at a threshold of *p* < 0.05, with false discovery rate (FDR) correction applied to account for multiple comparisons. To facilitate interpretation at the region of interest level, each electrode location was referred to the brain region it largely records from. For instance, the connectivity from AF3 to C3 was labeled as a Left Prefrontal (PFC) → Left Sensorimotor (SM) GC index, the GC value from P4 to C4 was labeled as a Right Parietal → Right Sensorimotor (SM) GC index, and the GC value from AF3 to P3 was labeled as a Left Prefrontal (PFC) → Left Parietal GC index. This approach was applied to all directional connections within and between hemispheres (mainly sensorimotor regions), providing a comprehensive measure of functional connectivity while preserving both directional information flow and hemispheric lateralization across cortical networks.

### 2.7. Statistical Analyses

Given the small sample size (*n* = 14) of participants who completed all 18 sessions, nonparametric statistical methods were applied. Within-group differences across time (Pre, Mid, and Post) were analyzed using the Friedman test for repeated measures, followed by Wilcoxon post hoc testing. Between-group comparisons were conducted using the Mann–Whitney U test. Benjamini–Hochberg false discovery rate (FDR) procedure was applied to account for multiple comparisons across EEG connectivity measures and post hoc analyses. Both unadjusted *p*-values and FDR-adjusted *q*-values are reported, with statistical significance defined as *q* < 0.05 after correction. Effect sizes (r) were calculated using the Wilcoxon Z statistic for pairwise comparisons and interpreted according to conventional thresholds (small = 0.10, moderate = 0.30, large ≥ 0.50). Continuous variables were reported as mean ± SD or median [IQR]. All statistical analyses were conducted using Python (version 3.8.8) [27].

## 3. Results

All participants completed the required study procedures without reporting any adverse events.

### 3.1. Right Hemisphere Functional Connectivity

**Figure 2:**
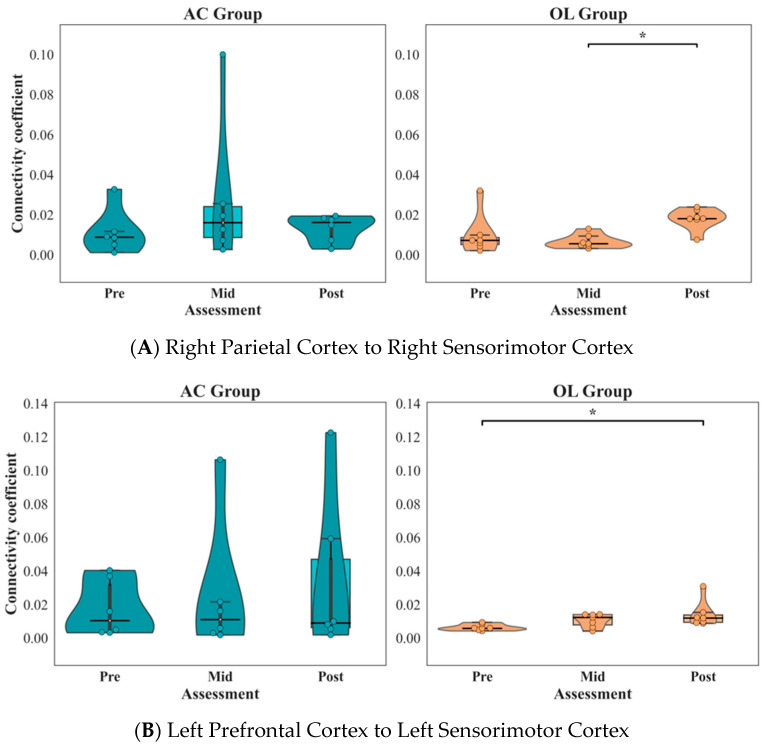
Longitudinal changes in functional connectivity (**A**) between right parietal cortex and right sensorimotor cortex and (**B**) left prefrontal cortex and left sensorimotor cortex following training. Violin plots depict the distribution of connectivity coefficients at pre-intervention, mid-intervention, and post-intervention for the AC and OL training groups. The overlaid boxplots indicate the median and interquartile range, while individual data points represent single participants. Horizontal bars and asterisks denote statistically significant FDR-adjusted differences (*p* < 0.05).

**Figure 3:**
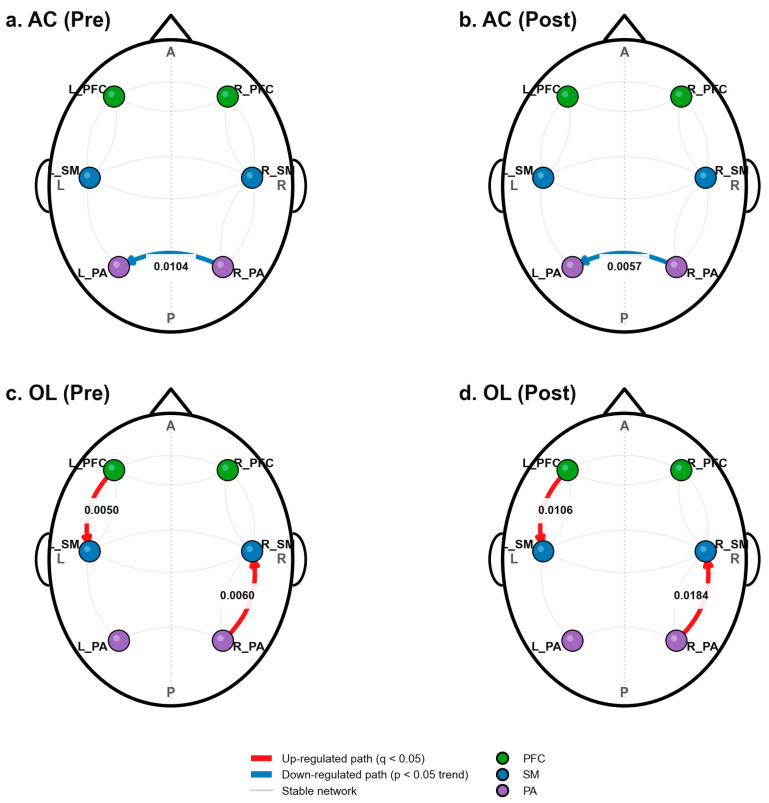
Task-related functional connectivity changes following training. Brain connectograms illustrating the longitudinal adaptations in resting-state functional connectivity for (**a**,**b**) the Active Control (AC) group and (**c**,**d**) the Offset Loading (OL) group. (**a**,**c**) display baseline (Pre) network configurations for each group. (**b**) shows a non-significant decrease (blue arrow) in task-related connectivity in the AC group, specifically from the right parietal area (R_PA) to the left parietal area (L_PA). (**d**) highlights significant training-induced increases (red arrows) in connectivity within the OL group, emerging on the trained left hemisphere from the prefrontal cortex (L_PFC) to the sensorimotor cortex (L_SM) and on the right hemisphere from the parietal area (R_PA) to the sensorimotor cortex (R_SM) (*q* < 0.05). Faded grey curves represent stable baseline network connections that did not change significantly. Color-coded spherical nodes represent distinct brain regions (metallic green: PFC, metallic blue: SM, metallic purple: PA). Numbers on the arrows indicate the median functional connectivity coefficients. A: Anterior, P: Posterior, L: Left, R: Right.

The OL group demonstrated a significant increase in functional connectivity coefficients from the right parietal to the right SM cortices following asymmetrical training (Friedman χ^2^ = 8.00, *p* = 0.018, FDR *q* = 0.037; Table 1, Figure 2 and Figure 3). Post hoc Wilcoxon signed-rank tests with FDR correction indicated a significant increase between Mid and Post (*p* = 0.0156, *q* = 0.0469, *r* = 0.894), with median connectivity increasing from 0.0060 [0.0048–0.0095] to 0.0184 [0.0176–0.0221]. No other pairwise comparisons remained statistically significant after FDR correction (all *q* > 0.05). Conversely, the AC group exhibited a significant omnibus effect for connectivity from the right PA to right FC cortices (Friedman *χ*^2^ = 7.00, *p* = 0.030, FDR *q* = 0.060), although post hoc Wilcoxon tests following FDR correction did not identify any sustained pairwise differences (all *p* > 0.05). Between-group differences were not significant at any time point (all *p* > 0.05).

### 3.2. Left Hemisphere Functional Connectivity

The OL group demonstrated a significant increase in functional connectivity coefficients from the left PFC to the left SM cortices following asymmetrical training (Friedman *χ*^2^ = 8.00, *p* = 0.018, FDR *q* = 0.037, Table 1; Figure 2). Post hoc Wilcoxon signed-rank tests with FDR correction indicated that this increase was driven by the Pre vs. Post comparison (*p* = 0.0156, *q* = 0.0469, *r* = 0.894, Table 1; Figure 2), and changes at the midpoint of the intervention (Pre vs. Mid: *p* = 0.0312, *q* = 0.0469, *r* = 0.831, Table 1; Figure 2), suggesting that connectivity adaptations emerged after sustained offset loading. Median connectivity increased from 0.0050 [0.0048–0.0089] at pre to 0.0106 [0.0094–0.0160] at post. In contrast, the AC group exhibited no significant changes in left hemispheric connectivity across time (Friedman *χ*^2^ = 0.33, *p* = 0.847). The between-group comparisons were not significant (all *p* > 0.05) due to high inter-individual variability.

### 3.3. Between-Hemispheres Functional Connectivity

Analyses of between-hemisphere connectivity revealed no significant longitudinal changes in the OL group and the AC group between left and right SM (all *p* and FDR *q* > 0.05; Table 1).

## 4. Discussion

This study shows that six weeks of upper extremity OL leads to specific changes in brain networks involving the prefrontal, parietal, and sensorimotor cortical regions in neurotypical adults. Using EEG-based functional connectivity analyses, we found OL increased the strength of connection from the left prefrontal cortex to the left sensorimotor cortex, suggesting enhanced cognitive control of upper extremity chest press movements. OL also increased the strength of connection from the right parietal cortex to the right sensorimotor cortex, suggesting enhanced sensory integration for the performance of the chest press movements. In contrast, six weeks of symmetrical resistance training failed to cause any systematic changes in brain networks. Our findings suggest that six weeks (or 18 training sessions) of OL stimulus was effective in altering brain networks related to cognitive and sensorimotor behavior.

### 4.1. Offset Loading and Cortical Network Reorganization

The prefrontal cortex is crucial for planning movements, controlling attention, and regulating actions, especially when tasks are challenging or when movements are unfamiliar or unstable [4]. Offset loading disrupts mechanical symmetry, which requires continuous attention to monitor limb movements, detection of errors caused by asymmetry, and ongoing adjustments. These demands are likely to keep the prefrontal cortex highly engaged during training [28,29]. In our study, OL training significantly increased the flow of information from the left prefrontal cortex to the left sensorimotor cortex. The left-lateralized prefrontal-sensorimotor connectivity enhancement likely reflects the left hemisphere’s dominant role in motor planning, sequencing, and predictive control, which is independent of hand dominance [30]. By contrast, the active control group showed no change in the connectivity patterns. This increase in information flow from the frontal to the central brain regions aligns with enhanced engagement of cognitive/executive processes for better control of upper extremity movements during asymmetric loading [31,32].

OL also improved the directed connectivity from the right parietal cortex to the right sensorimotor cortex. The right-lateralized parietal-sensorimotor connectivity enhancement, despite bilateral training exposure, reflects the right hemisphere’s specialized role in visuospatial processing, body schema representation, and online sensory-motor integration [31]. The parietal cortex is key for integrating body position information, understanding space, and converting sensory data into movement signals [32]. Asymmetric loading likely increases differences between limbs, which heightens the need for effective communication between the parietal and sensorimotor areas to maintain coordinated movement and body balance [33]. Our results indicate that this effect primarily developed following the third to sixth week of OL training (i.e., Mid to Post), suggesting that repeated exposure to OL gradually strengthens sensory and motor networks, which follows the principles of use-dependent brain changes [34].

Interhemispheric connectivity between left and right sensorimotor regions did not change significantly in either group. The lack of interhemispheric modulation implies that offset loading-induced plasticity was not due to increased bilateral coupling or compensatory recruitment across hemispheres. Instead, it resulted from the selective refinement of task-relevant cortical pathways within the hemisphere. These findings indicate that offset loading promotes specific cortical network adaptations rather than broad increases in global connectivity. This targeted reorganization differs from the generalized connectivity changes seen after aerobic or purely cognitive training and supports viewing offset loading as a task-specific cortical stimulus [8].

### 4.2. Comparison with Traditional Resistance Training

While traditional resistance training is known to induce neural adaptations during incipient stages of strength acquisition, these adaptations typically involve changes within primary motor regions and intracortical circuits [6,8]. In our study, there were no significant longitudinal changes in functional connectivity across left or right cortical pairs. The absence of palpable effect may be due to the relatively small sample size of the study, which limits statistical power to detect subtle neural changes when present. Also, the six-week intervention duration may be insufficient to elicit meaningful, measurable adaptations, as prior studies have shown that longer-duration training or higher-intensity models may be needed to observe robust neuroplastic effects in conventional resistance training [6]. However, much of the existing research has focused on symmetrical loading strategies and localized effects in the cortex. The current findings build on this research by showing that OL leads to changes in higher-order cortical networks involving the prefrontal and parietal regions. This highlights how task complexity and mechanical asymmetry drive central changes [17,34]. The results align with evidence that new, unstable, or cognitively demanding motor tasks activate a wider and stronger area in the cortex compared to repetitive, symmetrical exercises [19]. Practically, OL might be an effective and scalable way to improve cortical stimulation without raising the overall training volume [28,33].

### 4.3. Limitations and Future Directions

Several limitations of our study should be noted. First, the small sample size comes from the demanding nature of EEG data collection and supervised training, which may affect both statistical power and generalizability. Even though suitable nonparametric statistical methods were applied, larger randomized studies are needed to confirm these results and examine differences in cortical adaptation among individuals. Second, the participants were young, healthy, and mostly right-handed; thus, further studies are needed to understand the effects of OL on the brain networks in older adults, clinical groups, or those with marked neuromuscular imbalances. It should be noted that the sensor-level resolution is a limiting factor in determining the spatially specific activity within the selected prefrontal, central, and parietal electrodes due to volume conduction. Lastly, future work must study the behavioral implications of cortical reorganization following OL.

## 5. Conclusions

OL selectively increased functional connectivity from left prefrontal to left sensorimotor and right parietal to right sensorimotor cortices, reflecting enhanced cognitive control and sensorimotor integration. Symmetrical resistance training produced no significant changes, highlighting the task-specific nature of asymmetric resistance training for cortical plasticity. These findings may provide insights into how asymmetric resistance training alters neural and cognitive function, thereby informing future strategies for neurorehabilitation, cognitive enhancement, and injury prevention.

## Figures and Tables

**Table 1:** Longitudinal changes in EEG-directed functional connectivity between cortical regions in OL and AC training groups.

Cortical Pair	Group (*N* = 14); AC: 6, OL: 8	Pre Median [IQR]	Mid Median [IQR]	Post Median [IQR]	Friedman χ^2^	*p*-Value	FDR *q*	Pre–Mid (*p*, *q*, *r*)	Mid–Post (*p*, *q*, *r*)	Pre–Post (*p*, *q*, *r*)
Left SM → Left PFC	AC	0.0066 [0.0037–0.0186]	0.0159 [0.0038–0.0268]	0.0059 [0.0051–0.0287]	4.333	0.1146	0.1146	0.3125, 0.4688, 0.471	0.6875, 0.6875, 0.214	0.2188, 0.4688, 0.556
	OL	0.0062 [0.0040–0.0217]	0.0092 [0.0050–0.0119]	0.0139 [0.0091–0.0244]	4.571	0.1017	0.1146	0.9375, 0.9375, 0.064	0.0781, 0.2344, 0.703	0.2969, 0.4453, 0.447
Right PA → Right SM	AC	0.0066 [0.0029–0.0111]	0.0141 [0.0056–0.0220]	0.0128 [0.0052–0.0179]	2.333	0.3114	0.3114	0.0625, 0.1875, 0.813	0.5625, 0.5625, 0.300	0.5625, 0.5625, 0.300
	OL	0.0066 [0.0036–0.0095]	0.0060 [0.0048–0.0095]	0.0184 [0.0176–0.0221]	8.000	0.0183	0.0366	0.8125, 0.8125, 0.128	0.0156, 0.0469, 0.894	0.2969, 0.4453, 0.447
Left PFC → Left SM	AC	0.0104 [0.0034–0.0352]	0.0092 [0.0028–0.0216]	0.0077 [0.0037–0.0414]	0.333	0.8465	0.8465	0.4375, 0.6562, 0.385	0.4375, 0.6562, 0.385	0.6875, 0.6875, 0.214
	OL	0.0050 [0.0048–0.0089]	0.0127 [0.0062–0.0139]	0.0106 [0.0094–0.0160]	8.000	0.0183	0.0366	0.0312, 0.0469, 0.831	0.6875, 0.6875, 0.192	0.0156, 0.0469, 0.894
Left SM → Left PA	AC	0.0123 [0.0070–0.0151]	0.0099 [0.0060–0.0161]	0.0080 [0.0046–0.0106]	0.333	0.8465	0.8465	0.5625, 0.6875, 0.300	0.6875, 0.6875, 0.214	0.3125, 0.6875, 0.471
	OL	0.0067 [0.0025–0.0111]	0.0104 [0.0052–0.0126]	0.0161 [0.0075–0.0207]	3.714	0.1561	0.3122	0.9375, 0.9375, 0.064	0.1562, 0.4688, 0.575	0.4688, 0.7031, 0.319
Right SM → Right PFC	AC	0.0054 [0.0025–0.0183]	0.0054 [0.0017–0.0396]	0.0159 [0.0064–0.0276]	1.333	0.5134	0.5647	0.4375, 0.6562, 0.385	1.0000, 1.0000, 0.043	0.4375, 0.6562, 0.385
	OL	0.0021 [0.0019–0.0049]	0.0060 [0.0026–0.0079]	0.0070 [0.0051–0.0118]	1.143	0.5647	0.5647	0.6875, 0.6875, 0.192	0.3750, 0.6875, 0.383	0.6875, 0.6875, 0.192
Right PFC → Right SM	AC	0.0041 [0.0022–0.0150]	0.0143 [0.0038–0.0150]	0.0108 [0.0046–0.0173]	1.333	0.5134	0.5134	0.4375, 0.6562, 0.385	0.8438, 0.8438, 0.128	0.2188, 0.6562, 0.556
	OL	0.0094 [0.0051–0.0145]	0.0096 [0.0034–0.0160]	0.0092 [0.0059–0.0193]	2.571	0.2765	0.5134	0.5781, 0.8125, 0.256	0.1562, 0.4688, 0.575	0.8125, 0.8125, 0.128
Right SM → Right PA	AC	0.0080 [0.0034–0.0099]	0.0188 [0.0027–0.0344]	0.0123 [0.0066–0.0197]	4.333	0.1146	0.2291	0.2188, 0.3281, 0.556	0.8438, 0.8438, 0.128	0.0312, 0.0938, 0.899
	OL	0.0101 [0.0063–0.0162]	0.0176 [0.0080–0.0249]	0.0121 [0.0078–0.0159]	0.857	0.6514	0.6514	0.2188, 0.5625, 0.511	0.3750, 0.5625, 0.383	0.8125, 0.8125, 0.128
Left SM → Right SM	AC	0.0095 [0.0060–0.0108]	0.0101 [0.0050–0.0244]	0.0061 [0.0045–0.0200]	1.333	0.5134	0.5134	0.3125, 0.4375, 0.471	0.4375, 0.4375, 0.385	0.3125, 0.4375, 0.471
	OL	0.0066 [0.0044–0.0112]	0.0063 [0.0033–0.0094]	0.0302 [0.0146–0.0312]	2.000	0.3679	0.5134	0.2969, 0.4453, 0.447	0.0781, 0.2344, 0.703	0.4688, 0.4688, 0.319
Left PFC → Right PFC	AC	0.0089 [0.0034–0.0267]	0.0136 [0.0036–0.0396]	0.0113 [0.0060–0.0164]	0.087	0.9575	0.9575	0.5002, 1.0000, 0.471	0.6875, 1.0000, 0.214	1.0000, 1.0000, 0.043
	OL	0.0103 [0.0050–0.0160]	0.0149 [0.0057–0.0171]	0.0141 [0.0110–0.0185]	2.571	0.2765	0.5529	0.2188, 0.3281, 0.511	0.1562, 0.3281, 0.575	0.5781, 0.5781, 0.256
Left PA → Right PA	AC	0.0074 [0.0038–0.0117]	0.0148 [0.0090–0.0247]	0.0130 [0.0030–0.0146]	0.087	0.9575	0.9575	0.5002, 1.0000, 0.471	0.6875, 1.0000, 0.214	1.0000, 1.0000, 0.043
	OL	0.0117 [0.0078–0.0145]	0.0119 [0.0060–0.0165]	0.0152 [0.0112–0.0243]	2.571	0.2765	0.5529	0.2188, 0.3281, 0.511	0.1562, 0.3281, 0.575	0.5781, 0.5781, 0.256
Right SM → Left SM	AC	0.0083 [0.0051–0.0100]	0.0111 [0.0068–0.0177]	0.0069 [0.0041–0.0582]	0.087	0.9575	0.9575	0.5002, 1.0000, 0.471	0.6875, 1.0000, 0.214	1.0000, 1.0000, 0.043
	OL	0.0075 [0.0056–0.0088]	0.0080 [0.0041–0.0116]	0.0102 [0.0058–0.0233]	2.571	0.2765	0.5529	0.2188, 0.3281, 0.511	0.1562, 0.3281, 0.575	0.5781, 0.5781, 0.256
Right PFC → Left PFC	AC	0.0116 [0.0055–0.0321]	0.0112 [0.0041–0.0216]	0.0104 [0.0056–0.0211]	0.333	0.8465	0.8465	1.0000, 1.0000, 0.043	1.0000, 1.0000, 0.043	0.8438, 1.0000, 0.128
	OL	0.0100 [0.0051–0.0175]	0.0101 [0.0062–0.0145]	0.0105 [0.0054–0.0153]	1.000	0.6065	0.8465	1.0000, 1.0000, 0.043	0.3125, 0.8438, 0.471	0.5625, 0.8438, 0.300
Right PA → Left PA	AC	0.0104 [0.0053–0.0132]	0.0168 [0.0086–0.0301]	0.0057 [0.0030–0.0118]	7.000	0.0302	0.0604	0.1562, 0.1562, 0.642	0.1562, 0.1562, 0.642	0.0312, 0.0938, 0.899
	OL	0.0083 [0.0024–0.0165]	0.0093 [0.0061–0.0181]	0.0149 [0.0041–0.0355]	3.000	0.2231	0.2231	0.4375, 0.6562, 0.385	0.8438, 0.8438, 0.128	0.4375, 0.6562, 0.385

Values of the EEG connectivity coefficient are presented as median [interquartile range, IQR]. → represents direction; AC, active control; OL, offset loading (AC *n* = 6; OL *n* = 8); PFC, prefrontal cortex; SM, sensorimotor cortex; PA, parietal cortex. Within-group differences across time (Pre, Mid, Post) were assessed using the Friedman test, with Benjamini–Hochberg FDR correction across cortical pairs. Post hoc pairwise comparisons used Wilcoxon signed-rank tests with FDR adjustment. Effect size (r) was calculated from the Wilcoxon Z statistic (r = |Z|/√n); where values ~0.1, 0.3, and ≥0.5 indicate small, medium, and large effects, respectively. Two-sided *p* < 0.05 was considered statistically significant.

## Data Availability

Data used for this study will be provided upon reasonable request.
